# Maternal Health Beyond Delivery: action to address multifactorial health inequity

**DOI:** 10.1016/j.eclinm.2023.102348

**Published:** 2023-12-06

**Authors:** Edward Kwabena Ameyaw, Pascal Agbadi, Angela Dawson

**Affiliations:** a School of Graduate Studies and Institute of Policy Studies, Lingnan University, Hong Kong SAR, China; b L & E Research Consult Ltd, Wa, Ghana; c Department of Sociology and Social Policy, Lingnan University, Hong Kong SAR, China; d Faculty of Health, School of Public Health, University of Technology Sydney, NSW 2007, Australia

While significant gains have been made to address the global burden of maternal mortality, as demonstrated by the 34.5% reduction between 2000 and 2020,^1^ progress has been uneven. A disproportionate burden is evident in low-income and middle-income countries (LMICs),^1^^,^^2^ particularly in the perinatal phase,^3^, ^4^, ^5^ a neglected yet critical period for action to prevent morbidity and mortality. This series: ***Maternal Health in the Perinatal Period and Beyond***, jointly published in *The Lancet Global Health* and *eClinicalMedicine*, presents four papers outlining the complex factors driving adverse health outcomes in the perinatal and later post-partum periods and strategies to address this maternal inequity.

The first paper in this Series sets the scene by emphasising the multifaceted nature of maternal health and the need for comprehensive, socially informed interventions.^6^ In addition to biomedical factors, the paper highlights the need for researchers and policymakers to prioritise social and systemic changes to improve maternal health outcomes. The paper reveals the complex connections between socioeconomic factors, healthcare quality, and maternal mortality, emphasising the need for tailored, evidence-based strategies at national and regional levels. More importantly, the paper underscores the central role of the health system as a protective factor and accentuates the necessity of quality care in mitigating adverse maternal health outcomes. Paper two elaborates on the determinants of maternal health to explore vulnerabilities at the individual and community level affecting maternal health, emphasizing the dynamic and interconnected nature of these factors.^7^ Late pregnancy vulnerabilities, such as nutritional deficiencies, maternal depression, socioeconomic disparities, and environmental crises, significantly impact maternal and perinatal outcomes. The authors argue that it is important to understand how threats, barriers, and reparative strategies interact to augment policies and clinical practices to mitigate vulnerabilities during pregnancy, childbirth, and the postpartum period.

The third paper in the series underscores the critical gap in addressing medium-term and long-term complications after childbirth, shifting the focus beyond reducing maternal mortality to improving the overall wellbeing of postpartum women.^8^ This paper illustrates how biological, social, and clinical factors interact in complex ways to impact health and wellbeing, stressing the need for personalised, woman-centred care models. The lack of comprehensive data, especially in LMICs, raises concerns about the under-reporting and under-recognition of adverse conditions, highlighting the need for robust epidemiological studies. The paper makes the case that it is difficult to implement evidence-based practices because there are few high-quality guidelines, especially in LMICs. Improving maternal health will require reviewing existing guidelines to ensure that they are fit-for-purpose and can be immediately implemented at scale. The authors urge policymakers, health providers, and researchers to reconsider postpartum care models, expand the care timeframe beyond the standard six weeks, and engage multi-disciplinary teams in the provision of holistic postpartum care.

Finally, the fourth paper emphasises the need for a comprehensive approach to maternal health, that considers intersectional gender power relations.^9^ The authors advocate for an understanding of maternal health in relation to individuals’ social and political identities that affect discrimination and privilege and therefore advantage and disadvantage. These identities may result in racism, ethnic or caste-based discrimination, and gendered power relations. Using an intersectional lens, originating from Black feminist theory, the authors demonstrate how different types of power affect women's health in inequitable ways, limiting their ability to make choices, obtain resources, and avoid abuse during childbirth. The consideration of intersectionality and its application in maternal health analysis marks a crucial step toward more equitable and just healthcare systems for all. This perspective is pivotal for creating inclusive policies, practices, and research methodologies to tackle maternal health disparities.

Despite the great contribution of this Series to the discourse on maternal health, the authors raise several issues for reflection, debate, and research attention. Firstly, ending preventable maternal death and disability might require a reframing of the postpartum period beyond six weeks to ensure the mobilisation and investment of required resources. If so, then what will be the ideal period? Secondly, considering that vulnerabilities dominate in LMICs, should there be a different postpartum time frame to guide actions in LMICs than in high-income countries? As the Series has drawn our attention to the critical role of non-biomedical factors, there is a need for the research community to ascertain clearly defined parameters for measuring how these factors, independently or jointly, impact maternal conditions and their intensity. Of equal significance and warranting additional theoretical and empirical investigation, is the subjective perception, comprehension, and interpretations of women regarding their vulnerabilities. These issues are crucial in planning and implementing strategies to save lives.

## Contributors

EKA, PA, and AD were responsible for writing and editing the commentary.

## Declaration of interests

We have no conflict of interest.
